# Digital speech‐based markers to advance prognosis in Alzheimer's disease

**DOI:** 10.1002/alz.71489

**Published:** 2026-06-19

**Authors:** Melissa Lee, Stephan Meylan, Eli Shobin, the SpeechDx Consortium, Laura Nisenbaum, Aishwarya Sukumar, Lampros Kourtis

**Affiliations:** ^1^ Alzheimer's Drug Discovery Foundation New York New York USA; ^2^ Department of Linguistics University of California, Berkeley Berkeley California USA; ^3^ Gates Ventures Kirkland Washington USA

**Keywords:** Alzheimer's disease, artificial intelligence, biomarkers, blood biomarkers, cognition, cognitive impairment, cognitive markers, dementia, diagnosis, digital markers, disease monitoring, prognosis, prognostic biomarkers, speech

## Abstract

Validated prognostic tools are essential to advance drug development and clinical care in Alzheimer's disease, particularly as the field shifts toward the prevention of cognitive decline. While progress has been made in developing blood‐based biomarkers for the early detection of amyloid pathology, amyloid positivity alone does not reliably predict progression to symptomatic disease. Digital markers can serve as complementary prognostic tools to inform early intervention strategies. Among digital markers, speech‐based markers offer a scalable, non‐invasive, and cost‐effective approach to predicting and monitoring cognitive decline. However, the development of validated speech‐based tools has been constrained by the lack of large, multilingual datasets with longitudinal sampling, deep phenotyping, harmonized clinical and biomarker data, and adequate representation of preclinical populations. SpeechDx is a 3‐year, multinational, multilingual observational study (*n* = 2006) designed to address these gaps and accelerate the development of speech‐based tools to inform early risk assessment, enable timely intervention, and guide personalized care.

## Highlights

- Speech is a promising digital marker for prognosis in AD due to its real‐world feasibility and evidence of clinical utility.
- Speech offers a valuable complement to blood biomarkers for predicting future cognitive decline.
- Progress in developing speech‐based tools has been constrained by the absence of large, longitudinal, and deeply phenotyped datasets.
- SpeechDx is a 3‐year, multilingual observational study that will enable the development of diagnostic and prognostic tools for AD and related dementias.

## 1 INTRODUCTION

The prevalence of Alzheimer's disease (AD) is increasing rapidly, contributing to a growing public health crisis and demand for scalable approaches to early detection and intervention.^1^, ^2^, ^3^, ^4^ The need for timely diagnosis has become even more pressing with the advent of disease‐modifying therapies (DMTs) for AD, which are most effective when initiated early in the disease course.^5^, ^6^ Historically, early diagnosis has been limited by the absence of accessible diagnostic tools suitable for primary care – the first and sometimes only point of contact with the healthcare system for many people.^1^, ^3^, ^7^, ^8^ The recent emergence of AD blood‐based biomarkers marks a significant advancement in the diagnostic landscape, offering a less invasive, scalable alternative to traditional modalities such as cerebrospinal fluid (CSF) analysis and positron emission tomography (PET) imaging, which are often inaccessible outside of specialty care settings. These blood tests detect amyloid pathology, a core pathological hallmark of the disease.^7^, ^9^, ^10^ However, their prognostic value – the ability to predict future disease progression – is limited, as many people with amyloid accumulation remain asymptomatic for years or never develop clinical symptoms.^11^, ^12^ This raises concerns about overtreatment or suboptimal management, especially as DMTs that target amyloid pathology move into preclinical stages of the disease.^13^, ^14^, ^15^, ^16^ Recent findings demonstrating the potential efficacy of DMTs in these early stages underscore the urgency of developing scalable prognostic tools to help ensure that emerging treatments reach the right individuals at the right time.

**FIGURE 1:**
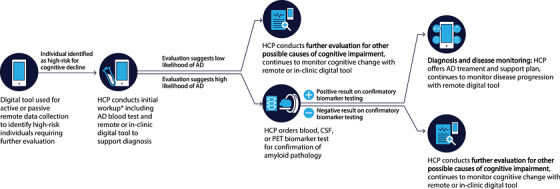
Potential integration points for digital markers in the AD patient pathway. This pathway begins with a remotely administered digital tool that identifies an individual's risk for cognitive decline based on longitudinal scoring of speech and language use. Digital markers are then integrated throughout the diagnostic process as a complement to biomarker testing, supporting a more timely and accurate diagnosis. If a diagnosis of cognitive impairment is confirmed, digital tools can be used for ongoing disease monitoring. If the diagnostic work‐up suggests cognitive impairment is unlikely, digital tools can be used to track cognitive health over time. *Workup may include a detailed history, physical exam, standard laboratory tests, evaluation of reversible causes of cognitive impairment, and neuroimaging (e.g., MRI/CT scan). AD, Alzheimer's disease; CI, cognitive impairment; CSF, cerebrospinal fluid; CT, computed tomography; HCP, healthcare professional; MRI, magnetic resonance imaging; PET, positron emission tomography.

Digital markers (defined as objective measures derived from digitally acquired data) offer a valuable complement to blood‐based biomarkers.^17^ When validated as functional measures of cognition (defined as a measure that reflects an individual's ability to function in a community), digital markers can support multiple use cases across the AD continuum – including prognosis (Figure 1). In addition, their non‐invasive, low‐cost, and remote deployment potential makes them highly scalable and well positioned to support more equitable access to intervention, including in underserved or geographically remote populations. Among digital markers, speech has emerged as a particularly promising functional measure of cognition, given its real‐world feasibility and the mounting evidence supporting its utility in AD.^18^, ^19^, ^20^, ^21^, ^22^, ^23^, ^24^

Despite this promise and despite major advances in natural language processing, machine learning, and artificial intelligence (AI) in recent years, the integration of speech‐based tools into clinical and research settings remains limited. A key barrier is the lack of high‐quality, longitudinal datasets linking speech features to clinical outcomes and biomarker‐defined measures of disease, especially in the preclinical stages of AD.^25^ These longitudinal datasets are necessary to train AI models to identify potential disease progressors. In this perspective, we argue that speech‐based markers represent a critical and underutilized opportunity to improve and scale the AD prognostic, diagnostic, and care pathway. We also introduce a study framework designed to address current data gaps and accelerate the development of validated speech‐based tools for cognitive impairment detection and prediction by enabling multilingual data collection, dense longitudinal sampling, linkage to biomarker‐confirmed diagnoses, and harmonized multimodal phenotyping across the disease continuum.

## 2 IMPLEMENTING SPEECH‐BASED MARKERS ACROSS THE AD CONTINUUM

Speech and language production are complex cognitive functions that rely on distributed networks of interconnected brain regions, which are frequently disrupted in AD. A growing body of research suggests that speech can provide an early and sensitive window into neurodegenerative processes associated with the disease.^18^, ^19^, ^24^ Acoustic and linguistic features of voice accurately distinguish individuals with AD or mild cognitive impairment (MCI) from cognitively normal controls.^21^, ^26^ These features also correlate strongly with structural and biomarker‐defined measures of AD burden, such as hippocampal atrophy and amyloid load.^22^, ^26^

Importantly, the ease of speech data capture, advances in AI, machine learning, and natural language processing, and the proliferation of smartphones and voice‐enabled technologies make speech‐based tools uniquely suited for remote, high‐frequency sampling. With more than 6.7 billion smartphone subscriptions worldwide and mobile penetration exceeding 80% in many low‐ and middle‐income countries, these tools can be deployed at scale and support both active (user‐initiated tests) and passive (ambient, while performing everyday functions) data collection, enabling repeated, longitudinal assessments across diverse populations with minimal or no burden.^18^, ^27^, ^28^, ^29^ This creates an unprecedented opportunity to leverage everyday interactions for scalable, unobtrusive detection or prediction of cognitive decline and ongoing disease monitoring. By extending cognitive assessment beyond traditional clinical encounters, speech‐based markers have the potential to transform when and how AD is identified, tracked, and managed.

## 3 PROGNOSTIC VALUE OF SPEECH‐BASED MARKERS

Of the many ways speech can improve the AD care pathway, advancing speech‐based prognostic tools should be a top priority. Prognosis remains one of the field's most critical and difficult‐to‐fill gaps, as predicting future decline is often more difficult than detecting existing cognitive impairment or pathology. As secondary prevention trials of DMTs move forward – aimed at preventing or delaying symptom onset in individuals with biomarker‐confirmed amyloid pathology^13^, ^14^, ^15^, ^16^ – a critical question emerges: How do we determine who should receive pre‐emptive or preventive treatment, with high patient burden, substantial financial cost, onerous monitoring, and potential safety risks, when a significant portion of amyloid‐positive individuals may never progress to clinical symptoms?

Speech is a promising solution. Models built using specific linguistic features have been shown to forecast the onset of AD in cognitively unimpaired individuals, before clinical symptoms emerge, and may outperform traditional neuropsychological test scores and demographic models in their ability to predict progression to AD.^19^, ^23^ Given the scientific evidence for its prognostic value and the ease of collection, developing speech‐based tools for prognostic assessment represents a compelling path forward. Moreover, with thoughtful implementation strategies that promote equitable access, these tools can also help reduce persistent healthcare disparities in early AD detection and care.^1^

## 4 LACK OF DATA IS A CENTRAL BARRIER TO DEVELOPING DIGITAL SPEECH‐BASED TOOLS FOR AD

While recent advances in AI, machine learning, and natural language processing have markedly expanded our ability to extract meaningful information from speech, the development of validated speech‐based tools for AD remains constrained by a fundamental limitation: the lack of large, longitudinal, and deeply phenotyped datasets.^25^ This data scarcity is among the most significant barriers to advancing speech as a clinically useful tool for prognosis, diagnosis, and disease monitoring.

As summarized in Table 1, currently available speech datasets are often modest in size, lack standardized data collection protocols, and provide limited publicly available information about their composition, quality, and methods.^30^, ^31^, ^32^ They are also predominantly cross‐sectional, constraining model development to static use cases such as disease detection. With a few exceptions, cognitive status is often assigned without biomarker confirmation of AD, which introduces diagnostic uncertainty because cognitive decline may result from a variety of causes. In addition, most existing datasets are only in English, restricting generalizability across real‐world settings. These limitations significantly hinder the ability to train and validate robust speech‐based tools, such as classification and prediction models.

**TABLE 1:** Existing speech‐based datasets.

Dataset	Language	Indication	Size	Cross‐sectional or longitudinal	Tasks	Other data
DementiaBank–Pitt Corpus^33^	English	Dementia	*N* = 397	Cross‐sectional	Picture description	Demographics, cognitive status, transcript annotations, MMSE
DementiaBank–Delaware Corpus^34^, ^35^	English	Mild cognitive impairment	*N* = 70	Cross‐sectional	Picture description, story narrative, procedural discourse, personal narrative	Demographics, additional health data, cognitive–linguistic battery, coded transcripts
ADReSS (INTERSPEECH 2020)^31^	English	Alzheimer's disease	*N* = 156	Cross‐sectional	Picture description	MMSE, timestamped transcripts
ADReSSo (INTERSPEECH 2021)^32^	English	Alzheimer's disease	AD and MMSE prediction set: 237 audio files; prediction of cognitive decline set: 105 audio recordings	Cross‐sectional, year‐2 follow‐up labels	Picture description, semantic/category fluency	Demographics, MMSE, cognitive status, diarized vocalization sequences, cognitive decline/no‐decline labels
ADReSS‑M (ICASSP 2023 SPGC)^36^	English, Greek	Alzheimer's disease	English training set *N* = 237; Greek test set *N* = 46	Cross‐sectional	Picture description	Demographics, MMSE, cognitive status
TAUKADIAL (INTERSPEECH 2024 Challenge)^37^	English, Chinese	Mild cognitive impairment	*N* = 387	Cross‐sectional	Picture description	Demographics, text transcripts, cognitive status for subset
Carolinas Conversations Collection (CCC)^38^	English	Aging	*N* = 250	Cross‐sectional	Natural conversations	Demographics, transcriptions, rich discourse context
ADNI4^39^, ^40^	English	Mild cognitive impairment, dementia, Alzheimer's disease	*N* = 465	Longitudinal	Story recall	Demographics, health data, cognitive status, cognitive assessments, neuroimaging, biofluid biomarkers, genetic data
Bridge2AI‑Voice (PhysioNet)^41^	English	Respiratory disorders, voice disorders, neurological disorders, mood disorders, pediatric	*N* = 833	Cross‐sectional	Sustained phonation	Demographic information, health questionnaires, targeted questionnaires inquiring about known confounders for voice, disease specific information
VoxAging^42^	English, Mandarin	Aging	*N* = 293	Longitudinal, up to 17 years	Speech sourced from YouTube and Bilibili	Demographics
EARS (Elderly Adults Read Speech)^43^	Japanese	Aging	*N* = 123	Cross‐sectional	Read speech	Demographics, health data
Framingham Heart Study (subset)^44^, ^45^	English	Aging	*N* = 4293	Longitudinal	Neuropsychological testing sessions (including tasks)	Demographics, cognitive status, neuroimaging, neuropsychological testing, additional health data
SIDE‐AD (Speech for Intelligent cognition change tracking and DEtection of Alzheimer's Disease)^46^	English	Mild cognitive impairment, dementia, Alzheimer's disease	*N* = 450	Longitudinal, up to 2 years	Prompt to talk about brain health	Demographics, self‐reported cognitive status, additional neuropsychological assessments and biomarker data for a subset of participants
SpeechDx	English, Spanish, Catalan	Mild cognitive impairment, dementia, Alzheimer's disease	*N* = 2006	Longitudinal, 3 years	Picture description, story recall, storytelling, open ended questions	Demographics, neuropsychological test battery, MRI, blood biomarkers, genetic data, additional health data, additional biomarker data for a subset of participants

Abbreviations: AD, Alzheimer's disease; MMSE, Mini‐Mental State Examination; MRI, magnetic resonance imaging.

The absence of longitudinal data is particularly challenging for prognostic applications. AD progresses gradually over many years,^47^ and models intended to predict or monitor cognitive decline must be trained on data that reflect this temporal evolution. Without repeated speech assessments linked to clinical and biomarker outcomes, it is not possible to build tools that accurately capture individual trajectories over time.

Longitudinal datasets that link repeated speech assessments with clinical and biomarker data across diverse populations and disease stages are critical to advance the field.^48^ Yet the financial and logistical burden of building such datasets – particularly those requiring deep phenotyping with repeated neuroimaging, fluid biomarkers, and neuropsychological characterization – makes these large‐scale efforts unfeasible for most research groups. Coordinated, multi‐institutional efforts and the creation of interoperable data platforms are needed, and momentum toward such efforts is building.^46^

## 5 LONGITUDINAL STUDY DESIGN FOR PROGNOSTIC SPEECH‐BASED TOOL DEVELOPMENT IN AD

To address the need for a multilingual, longitudinal dataset with densely sampled speech recordings and harmonized clinical and biomarker data, we designed SpeechDx, a 3‐year, multinational observational study (Table 1).^49^ The primary goal of the study is to establish a gold‐standard dataset that will enable the development of prognostic speech‐based tools that predict future cognitive decline in AD. The secondary aims are to support diagnostic and disease‐monitoring speech‐based marker development for Alzheimer's disease and related dementias (ADRD).

SpeechDx builds on established AD research cohorts that were selected based on compatibility of operational aims, with consideration of cohort characteristics, size, ongoing study compatibility, and language. Participant inclusion and exclusion criteria are intentionally minimal to maximize inclusivity and representativeness of a real‐world population. Inclusion criteria include age ≥45 years, access to 3G connectivity or better, and native proficiency in English, Spanish, or Catalan. Exclusion criteria include history of severe traumatic brain injury, severe depression, schizophrenia, stroke, or brain tumors; significant uncorrected vision or hearing impairment; or any neurological or psychiatric disorder that precludes completion of required assessments. While minimal criteria are a strength, we acknowledge that they introduce heterogeneity that must be addressed in downstream analyses. Participating research cohorts are responsible for implementing strategies to promote equity, minimize bias, and support participant retention within this heterogeneous study population.

### 5.1 SpeechDx is designed to enable earliest disease detection

SpeechDx has enrolled 2006 participants across sites in the United States, Australia, and Spain. A key design feature is longitudinal follow‐up to maximize the ability to capture transitions in disease state, biomarkers, and neuropsychological profiles across diagnostic stages. The study population spans the full cognitive spectrum – including cognitively normal individuals and those with subjective cognitive decline, MCI, and AD dementia – but is enriched for cognitively normal individuals and individuals with subjective cognitive decline to support the earliest possible detection of disease‐related changes and the development of prognostic tools.

### 5.2 Standardized speech data collection

**FIGURE 2:**
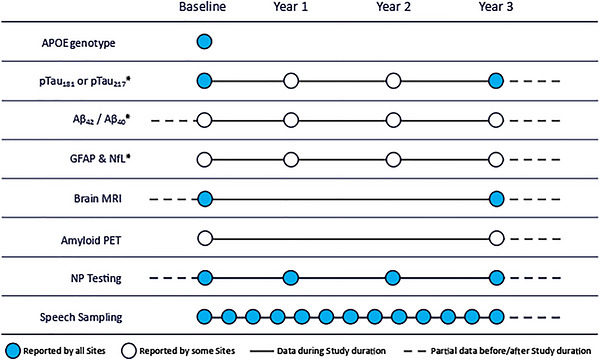
Timeline for speech, clinical, and biomarker data collection. Participants from all sites complete remote speech assessments every 3 months over the 3‐year study period. In‐person clinical assessments occur annually or every 18 months, depending on the protocols of their respective parent studies. Certain assessments are standardized across all sites (blue dots), while others may vary between sites (white dots). In‐person assessments include digital or traditional neuropsychological testing, genetic testing, MRI, PET imaging, and CSF and blood‐based Alzheimer's disease biomarker analysis. *Measured in plasma or CSF. Aβ, amyloid beta; APOE, apolipoprotein E; CSF, cerebrospinal fluid; GFAP, glial fibrillary acidic protein; MRI, magnetic resonance imaging; NfL, neurofilament light; NP, neuropsychological testing; PET, positron emission tomography; pTau_181_, tau phosphorylated at position 181; pTau_217_, tau phosphorylated at position 217.

Participants provide speech and language samples remotely once every 3 months over the 3‐year study period using a custom‐built SpeechDx application (app) designed for standardized, remote data capture (Figure 2). Each participant receives a study‐provided Android tablet (Samsung Tab A7 Lite) preloaded with the SpeechDx app. This standardized approach ensures consistency in device specifications, such as sensors and microphone quality, while also enhancing inclusivity by providing access to participants who may not own a compatible device.

Each assessment session consists of a structured battery of tasks designed to elicit semi‐naturalistic speech and capture contextual factors relevant to speech production (latent variables). Administration time is approximately 10 to 15 min for cognitively normal individuals. The battery includes picture description and recall tasks, open‐ended questions, story recall, and storytelling, alongside brief questionnaires (Patient Health Questionnaire‐8 [PHQ‐8] for mood and Karolinska Sleepiness Scale) and a psychomotor vigilance test assessing reaction time and sustained attention.^50^, ^51^, ^52^, ^53^, ^54^, ^55^, ^56^, ^57^, ^58^, ^59^ The assessment follows a standardized sequence beginning with picture description (using a new image at each session), the PHQ‐8 and Karolinska questionnaires, and three open‐ended questions, followed by picture recall and two story recall tasks, the vigilance test, a storytelling task, and a final picture description using a fixed image across sessions. The full assessment is designed to be completed in a single sitting with minimal participant burden, supporting repeated longitudinal sampling. Additional details regarding the rationale and operationalization of these tasks are available in the publicly available study procedures document (Section 3.2.2, p. 12; Section 4.8, pp. 22 to 29; Section 6, pp. 41 to 42).^49^

To support participant compliance, the app sends push notifications prompting completion of the assessment battery, supplemented by email reminders. Participants also receive advance notifications on their devices to alert them to upcoming scheduled assessments. Study site managers are advised to proactively contact participants to notify them of upcoming sessions and to follow up when assessments are not completed in a timely manner. Ultimately, responsibility for monitoring and managing participant compliance resides with the study sites, which can track adherence through a SpeechDx‐provided dashboard in the study portal that flags upcoming, overdue, and missed assessments.

To support the generalizability of speech measures across sites and populations, a primary design focus is ensuring linguistic applicability across the diverse participant population, which includes speakers of English, Spanish, and Catalan. Rather than relying on direct translations, which often results in unnatural or culturally unfamiliar wording, the app content is designed to be culturally neutral and linguistically appropriate across all three languages, with verification from native speakers. In addition, task stimuli, created in collaboration with the Boston University Framingham Heart Study team, are vetted by neuropsychologists to minimize bias in the pictures, stories, and prompts used.

### 5.3 Clinical characterization and harmonization of data from existing AD cohorts

Robust model development requires a well‐characterized dataset with biomarker‐confirmed diagnoses. To meet this requirement, SpeechDx leverages existing AD research cohorts to collect baseline cohort characterization data and longitudinal clinical and biomarker data, in parallel with longitudinal speech data collection (Figure 2).

Across participating sites, SpeechDx requires the collection of a core set of clinical, biomarker, and cohort descriptor variables. At baseline, participants are characterized using medical history, diagnosis, primary language, and additional demographic information (e.g., age, sex, race, ethnicity, and education), collected in alignment with the Uniform Data Set standards where possible and otherwise according to site‐specific protocols.^60^, ^61^ Baseline characterization also includes structural magnetic resonance imaging (MRI) (T1‐weighted and T2 fluid‐attenuated inversion recovery), blood‐based AD biomarkers (tau phosphorylated at threonine 181 [p‐tau181] or p‐tau217), and apolipoprotein E genotype. A neuropsychological test battery is available for most participants, though the specific tests may vary. This typically includes Digit Span, Category Fluency, Trail Making Test A/B, Verbal Fluency, and Montreal Cognitive Assessment (MoCA) or Mini‐Mental State Examination (MMSE). For a subset of study sites, baseline characterization also includes amyloid PET imaging and CSF biomarkers. Many of these clinical and biomarker measures are collected longitudinally over the study period, as available by site (Figure 2). Collectively, these multimodal clinical and biomarker data serve as the ground truth for training and validating prognostic and diagnostic models using the SpeechDx dataset.

A core component of the study is data harmonization across clinical sites. This process involves combining and centralizing data from various sources, data cleansing (removing duplicates or orphan entries), matching identifiers, normalizing measurements when possible, and aligning data fields to ensure consistency and compatibility across all sites. Additional details on the harmonization framework and quality‐control procedures are provided in the publicly available study procedures document (Section 4.9, pp. 33 to 37).^49^

### 5.4 Developing a unified dataset of speech and harmonized clinical and biomarker data

**FIGURE 3:**
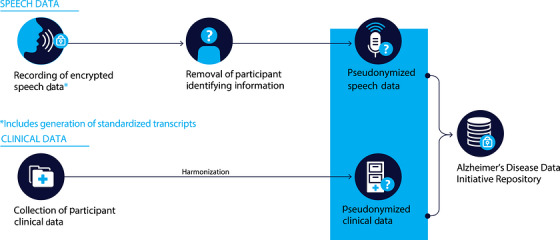
Workflow to establish a unified dataset for speech‐based AD research. All speech data are encrypted and securely uploaded to a cloud server. Transcripts are automatically generated and then manually reviewed to remove personally identifiable information and personal descriptors. Clinical and biomarker data from study sites and individual speech samples are harmonized across all study sites to create the unified SpeechDx dataset. Pseudonymized data are centralized in a data‐sharing repository managed by the AD Data Initiative's AD Curation Studio. Upon study completion, the full unified dataset will be hosted on the AD Curation Studio, where it will remain encrypted.

SpeechDx will generate ∼4000 h of high‐quality multilingual speech recordings, along with annotated transcripts and associated metadata (i.e., session date and time and standardized recording‐quality descriptors). The unified dataset will comprise audio files and transcripts linked to pseudonymized participant‐level characterization labels – including medical history, clinical diagnosis, language, demographic variables, neuropsychological test performance, imaging, and blood‐based biomarkers – harmonized across study sites (Figure 3; see Section 5.3 for additional details on cohort characterization).

Because voice recordings may inadvertently capture personally identifiable information (PII), voice recordings are encrypted and password‐protected, and access is limited to prevent direct playback. Trained International Organization of Standardization‐certified language professionals will manually review each recording, removing segments containing PII and replacing them with a tone of a specific frequency to indicate redaction. In parallel, each audio recording undergoes human‐corrected, automatic speech recognition‐based transcription to establish a ground‐truth record of speech content. As part of this standardized review process, SpeechDx implements a recording‐quality labeling framework in which each clip is systematically annotated using predefined quality descriptors (e.g., “silent” [no speech], “noisy” [background interference], “low volume” [insufficient speech levels]) to capture environmental and technical factors that may affect signal integrity. Additional annotations include speech disfluencies, such as pauses, unintelligible words, prolonged sounds or syllables, and incomplete word fragments. These metadata are available on the site dashboard to support clinical sites’ participant management and data quality oversight during the study. For additional details please refer to the publicly available study procedures document (Section 4.8.8, pp. 30 to 32).^49^

All pseudonymized data are centralized in a data‐sharing repository managed by the Alzheimer's Disease (AD) Data Initiative's AD Curation Studio – a secure, cloud‐based platform designed for data sharing and analytics (Figure 3).^62^ Upon study completion, the unified dataset will be hosted and encrypted on the AD Curation Studio, where researchers can access it within the secure computing environment. Users can apply their own bioinformatics tools to the dataset to build and refine machine learning and other AI models or use tools offered by AD Data Initiative, which supply the necessary computing power to support these analyses.

Importantly, longitudinal remote data collection over the 3‐year study period may be affected by participant‐level factors such as digital literacy, physical and mental health fluctuations, environmental noise, and technological barriers. These factors can influence data completeness and quality and may introduce algorithmic bias if not appropriately addressed during model development and validation. In addition, while the multilingual design of SpeechDx is a key strength, it also introduces linguistic variability that downstream developers will need to account for in model development.

## 6 CONCLUSIONS

With the Alzheimer's field rapidly advancing toward earlier detection and intervention, there is a growing need for tools that can identify individuals at high risk for cognitive decline. Recent innovations in AI, mobile health technologies, and blood‐based biomarkers have opened a window of opportunity to validate and scale digital markers designed to detect subtle changes in cognition. Among these, we argue that speech stands out as the most promising modality to fill this critical gap. High‐quality longitudinal datasets like SpeechDx are essential to support the development of speech‐based tools for prognosis, as well as to support diagnosis and disease monitoring. Looking ahead, sustained investment and cross‐sector collaboration will be key to advancing scalable digital markers that can inform early risk assessment, enable more timely diagnosis, and support personalized care – ultimately improving outcomes for individuals with or at risk for AD.

## AUTHOR CONTRIBUTIONS

All authors contributed to the conception and writing of the paper and provided critical feedback. M.L. was responsible for overall direction and planning.    

## CONFLICT OF INTEREST STATEMENT

Melissa Lee, Eli Shobin, and Laura Nisenbaum are employees of the Alzheimer's Drug Discovery Foundation. Stephan Meylan and Lampros Kourtis are paid consultants of the Alzheimer's Drug Discovery Foundation. Aishwarya Sukumar is an employee of Gates Ventures. The SpeechDx Consortium receives funding from the Alzheimer's Drug Discovery Foundation's Diagnostics Accelerator. Author disclosures are available in the supporting information.

## Supporting information



Supporting Information

## 1

SpeechDx Consortium contributors to be indexed to the paper.

**Table:**

Name	Affiliation
Yvonne Bannon	Psychiatry and Behavioral Neurosciences Memory Disorder Clinic, University of South Florida
Julianna Coura de Moura	Psychiatry and Behavioral Neurosciences Memory Disorder Clinic, University of South Florida
Yael Bensoussan	Department of Otolaryngology, University of South Florida
Jamie Toghranegar	Department of Otolaryngology, University of South Florida
Jacqueline Persaud	Department of Otolaryngology, University of South Florida
Ram Bishnoi	Psychiatry and Behavioral Neurosciences, University of South Florida
Joette Giovinco	Psychiatry and Behavioral Neurosciences, University of South Florida
Karim Hanna	USF Family Medicine, University of South Florida
Felicia Goldstein	Department of Neurology and Goizueta Alzheimer's Disease Research Center, Emory University School of Medicine
Allan Levey	Department of Neurology and Goizueta Alzheimer's Disease Research Center, Emory University School of Medicine
Sergi Valero	Ace Alzheimer Center Barcelona, Universitat Internacional de Catalunya; Networking Research Center on Neurodegenerative Diseases (CIBERNED), Instituto de Salud Carlos III
Andrea Miguel	Ace Alzheimer Center Barcelona, Universitat Internacional de Catalunya
Berta Calm	Ace Alzheimer Center Barcelona, Universitat Internacional de Catalunya
Lucy Mackintosh	The Florey Institute of Neuroscience and Mental Health
Joanne Robertson	The Florey Institute of Neuroscience and Mental Health
Christopher Fowler	The Florey Institute of Neuroscience and Mental Health
Colin Masters	The Florey Institute of Neuroscience and Mental Health
Stephanie Rainey‐Smith	Murdoch University
Kevin Taddei	Edith Cowan University
Ralph Martins	Macquarie University
Larry Ward	The Florey Institute of Neuroscience and Mental Health
Gabriele Cattaneo	Institut Guttmann, Institut Universitari de Neurorehabilitació adscrit a la UAB; Fundació Institut d'Investigació en Ciències de la Salut Germans Trias i Pujol
Javier Solana‐Sánchez	Institut Guttmann, Institut Universitari de Neurorehabilitació adscrit a la UAB; Fundació Institut d'Investigació en Ciències de la Salut Germans Trias i Pujol
Simon Fankhauser	Institut Guttmann, Institut Universitari de Neurorehabilitació adscrit a la UAB; Fundació Institut d'Investigació en Ciències de la Salut Germans Trias i Pujol; Departament de Medicina, Facultat de Medicina i Ciències de la Salut i Institut de Neurociències, Universitat de Barcelona
Edgar A. Buloz‐Osorio	Institut Guttmann, Institut Universitari de Neurorehabilitació adscrit a la UAB; Fundació Institut d'Investigació en Ciències de la Salut Germans Trias i Pujol
Gemma Albi‐Villuendas	Institut Guttmann, Institut Universitari de Neurorehabilitació adscrit a la UAB; Fundació Institut d'Investigació en Ciències de la Salut Germans Trias i Pujol
Marc Navarro‐Berenguel	Institut Guttmann, Institut Universitari de Neurorehabilitació adscrit a la UAB; Fundació Institut d'Investigació en Ciències de la Salut Germans Trias i Pujol
Álvaro Pascual‐Leone	Hinda and Arthur Marcus Institute for Aging Research and Deanna and Sidney Wolk Center for Memory Health, Hebrew SeniorLife, Harvard Medical School; Department of Neurology, Harvard Medical School
David Bartrés‐Faz	Institut Guttmann, Institut Universitari de Neurorehabilitació adscrit a la UAB; Departament de Medicina, Facultat de Medicina i Ciències de la Salut i Institut de Neurociències, Universitat de Barcelona; Institut d'Investigacions Biomèdiques August Pi i Sunyer (IDIBAPS)
Ileana De Anda‐Duran	Epidemiology Department, Celia Scott Weatherhead School of Public Health and Tropical Medicine, Tulane University
Lydia A. Bazzano	Epidemiology Department, Celia Scott Weatherhead School of Public Health and Tropical Medicine, Tulane University
Vanessa Fonseca	Epidemiology Department, Celia Scott Weatherhead School of Public Health and Tropical Medicine, Tulane University
Rhoda Au	Boston University Chobanian and Avedisian School of Medicine
Kristi Ho	Boston University Chobanian and Avedisian School of Medicine
Alexa Burk	Boston University Chobanian and Avedisian School of Medicine
Cody Karjadi	Boston University Chobanian and Avedisian School of Medicine
Andrew J. Saykin	Indiana Alzheimer's Disease Research Center and Department of Radiology and Imaging Sciences, Indiana University School of Medicine
Sarah Van Heiden	Indiana Alzheimer's Disease Research Center and Department of Radiology and Imaging Sciences, Indiana University School of Medicine
Christelle Elvinia	Indiana Alzheimer's Disease Research Center and Department of Radiology and Imaging Sciences, Indiana University School of Medicine
Fernanda Campos Strazzi	Barcelonaβeta Brain Research Center (BBRC), Pasqual Maragall Foundation
Lidia Canals‐Gispert	Barcelonaβeta Brain Research Center (BBRC), Pasqual Maragall Foundation
Alba Cañas‐Martínez	Barcelonaβeta Brain Research Center (BBRC), Pasqual Maragall Foundation
Jordi Freixa Canela	Barcelonaβeta Brain Research Center (BBRC), Pasqual Maragall Foundation
Sherezade Fuentes‐Julian	Barcelonaβeta Brain Research Center (BBRC), Pasqual Maragall Foundation
Pilar González‐Tartière	Barcelonaβeta Brain Research Center (BBRC), Pasqual Maragall Foundation
Laura Hernández Penas	Barcelonaβeta Brain Research Center (BBRC), Pasqual Maragall Foundation
Núria Lleonart i Camps	Barcelonaβeta Brain Research Center (BBRC), Pasqual Maragall Foundation
Carlota Medina Rodríguez	Barcelonaβeta Brain Research Center (BBRC), Pasqual Maragall Foundation
Xavier Meléndez Giner	Barcelonaβeta Brain Research Center (BBRC), Pasqual Maragall Foundation
Isabel Pérez Gutiérrez	Barcelonaβeta Brain Research Center (BBRC), Pasqual Maragall Foundation
Andreea Rădoi	Barcelonaβeta Brain Research Center (BBRC), Pasqual Maragall Foundation
Mireia Sánchez Gutiérrez	Barcelonaβeta Brain Research Center (BBRC), Pasqual Maragall Foundation
Pau Sánchez	Barcelonaβeta Brain Research Center (BBRC), Pasqual Maragall Foundation
Gonzalo Sánchez‐Benavides	Barcelonaβeta Brain Research Center (BBRC), Pasqual Maragall Foundation; Hospital del Mar Research Institute; Centro de Investigación Biomédica en Red de Fragilidad y Envejecimiento Saludable (CIBERFES)
Anna Soteras Prat	Barcelonaβeta Brain Research Center (BBRC), Pasqual Maragall Foundation
